# The Green Maternity project: A midwife‐led initiative to promote correct waste segregation on an Australian postnatal ward

**DOI:** 10.1111/jan.15789

**Published:** 2023-07-23

**Authors:** Vidanka Vasilevski, Jessica Huynh, Anna Whitehead, Ciara Noble, Carlos Machado, Linda Sweet

**Affiliations:** ^1^ School of Nursing and Midwifery Deakin University Burwood Victoria Australia; ^2^ Centre for Quality and Patient Safety Research Western Health Partnership, Institute for Health Transformation Geelong Victoria Australia; ^3^ Western Health St. Albans Victoria Australia

**Keywords:** carbon emissions, healthcare, medical waste disposal, midwife, waste management

## Abstract

**Aims:**

Healthcare waste production is a significant contributor to carbon emissions, negatively impacting the environment. Ineffective healthcare waste disposal results in greater measures to manage it which is costly to both the environment and healthcare organizations. This study aimed to improve waste management in a tertiary maternity hospital. Specifically, the impact of a midwife‐led intervention to improve waste segregation, staff knowledge and attitudes and waste management‐related costs was investigated.

**Design:**

A multi‐method study including pre‐ and post‐intervention staff waste management knowledge and attitude surveys and waste audits of bins located on the postnatal ward.

**Methods:**

The intervention included education sessions, posters and signage by waste bins and monthly newsletters distributed throughout 2021 to raise staff awareness of correct waste segregation processes. Pre‐ and post‐intervention surveys were distributed in early 2021 and early 2022, respectively. The waste audits occurred on three occasions, January, July and December of 2021. The waste audit included total waste in kilograms (kg), waste in kg by segregation and identification of correct and incorrect segregation. Waste audit and quantitative staff survey data were analysed using descriptive statistics and chi square. Qualitative data from the staff surveys were analysed using content analysis.

**Results:**

Knowledge and attitudes to waste management were similar across pre‐ and post‐intervention staff surveys. Knowledge of accurate allocation of specific items to waste streams was variable with errors identified in both the pre‐ and post‐surveys. Waste audit data showed reductions in clinical waste at each measurement, with a 71.2% decrease in clinical waste from baseline to the final audit. Accuracy of waste segregation also improved from the baseline to final audit, resulting in a 48% reduction in waste management costs.

**Conclusion:**

The midwife‐led initiative improved waste segregation and achieved the associated waste management cost reduction.

**Impact:**

A midwifery‐led initiative to address waste production and segregation on a maternity ward had a positive impact on waste segregation practices and associated waste management costs. The existence of change champions along with in‐service sessions, posters and newsletters to raise awareness of correct waste segregation resulted in a 71% reduction of incorrect items being placed in clinical waste bins. Challenges such as COVID‐19 pressures and workload made it difficult for midwives to engage in waste management education and effective waste segregation.

**Patient or Public Contribution:**

No patient or public contribution.

**What Does this Paper Contribute to the Wider Global Clinical Community?:**

Implementing clinician‐led waste management interventions across hospital wards while addressing workload issues are likely to have significant cost benefits for organisations and minimise the environmental impacts of healthcare settings.

## 1 INTRODUCTION

Carbon emissions have been categorized as Scope 1, 2 and 3. Scope 1 and 2 emissions refer those that are directly produced by an entity or indirectly produced to generate power used by an entity, respectively (Environmental Protection Authority, 2023). Scope 3 emissions are those that are indirectly produced as a result of an entity's use of goods and services (Swann, 2020). The Australian healthcare system contributes 7% of all carbon emissions, and the public hospital system is the highest contributor to this (Malik et al., 2018). The majority of these emissions are in the Scope 3 category. Focusing on the healthcare system should therefore be a priority for achieving Austrailia's carbon emission targets (Malik et al., 2018). With forecast increases in healthcare use (Goss, 2008), targeted action is required to reduce carbon emissions from the industry.

Carbon emissions contribute to climate change, increasing environmental temperatures, which can negatively impact health by increasing the risk of dehydration, heat stroke and respiratory problems (Sustainability Victoria, 2020). It has been argued that improper healthcare waste management and resulting emissions will significantly impact the global population, increasing pressure on public health services and resources in the future (Eckelman & Sherman, 2016).

### 1.1 Background

Healthcare services are a major contributor to environmental pollution (West et al., 2020; World Health Organization, 2018). An estimate of healthcare waste produced by all Australian healthcare services is difficult to obtain, however, a 2010–2011 report of public health system waste from the state of Victoria documented total waste volume at 42,000 tonnes (Victoria State Government, 2022). Of this volume, 8600 tonnes was recyclable, 4300 tonnes was clinical waste and the remaining was general waste (Victoria State Government, 2022).

Clinical waste includes sharps, medical waste (infectious or pathogenic), pharmaceuticals and radioactive materials. Managing clinical waste costs approximately $17 million per annum, with 60% of expenditure on effectively treating and disposing of clinical waste (Victoria State Government, 2022).

The inappropriate disposal of general waste into the clinical waste stream has considerable consequences on the environment, public health and healthcare service costs (Kagoma et al., 2012; Wyssusek et al., 2019). Clinical waste requires stringent management as it may be contaminated with human tissue or fluids that can potentially cause disease (Padmanabhan & Barik, 2019). Measures to manage clinical waste thus have the greatest environmental impact (Ghali et al., 2023). Healthcare providers have reported that clinical bins contained both general and clinical waste (Hames, 2013).Correct segregation of clinical versus general waste is an important strategy for ensuring that healthcare services minimize the use of clinical waste management measures. The effect of healthcare‐related waste on the environment and health service budgets can be dramatically reduced with appropriate waste identification and segregation at the source of initial disposal (West et al., 2020).

While sustainable healthcare practices are valued by clinicians (Ryan et al., 2020; Ward et al., 2022), several factors inhibit appropriate waste segregation in healthcare services. Poor staff habits, a perception that all waste is clinical waste, or that waste management is someone else's role have been identified (Hames, 2013; Wyssusek et al., 2019). Furthermore, inconvenient bin locations, confusion on whether an item is clinical or non‐clinical waste, and high patient turnover rates also contributed to waste disposal being a low priority for busy clinical staff (Department of Health and Human Services, 2018; Hames, 2013).

Healthcare staff have reported feeling inadequately educated in healthcare waste management (Elsayed & Gab‐Allah, 2019), which resulted in inefficient disposal of healthcare waste (Sustainability Victoria, 2020; Ward et al., 2022). A study by Sustainability Victoria highlighted a gap in healthcare professionals' understanding of waste management and the need for ongoing professional development training (Sustainability Victoria, 2020). Research has shown that with tailored education, clinical staff demonstrated increased knowledge in effective waste management (Elsayed & Gab‐Allah, 2019). Implementing interventions to support adequate waste segregation by staff can reduce operational costs, environmental impacts and risks to staff and patient health (World Health Organization, 2022; World Health Organization, Regional Office for Europe, 2017).

## 2 THE STUDY

### 2.1 Aim

This study aimed to improve waste management in a tertiary maternity hospital in the western suburbs of Melbourne, Victoria. Specifically, the impact of a midwife‐led intervention to improve waste segregation, staff knowledge and attitudes, waste volume and waste management‐related costs was investigated.

#### 2.1.1 The ‘Green Maternity’ intervention

**FIGURE 1:**
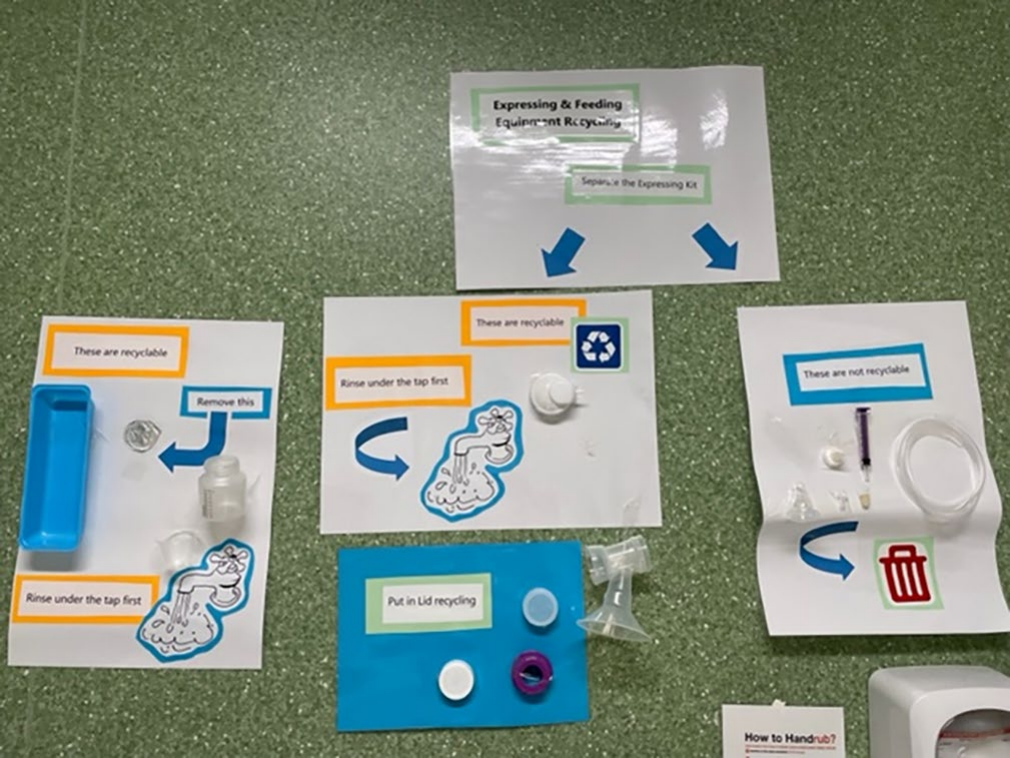
Example posters used to inform correct waste segregation.

**FIGURE 2:**
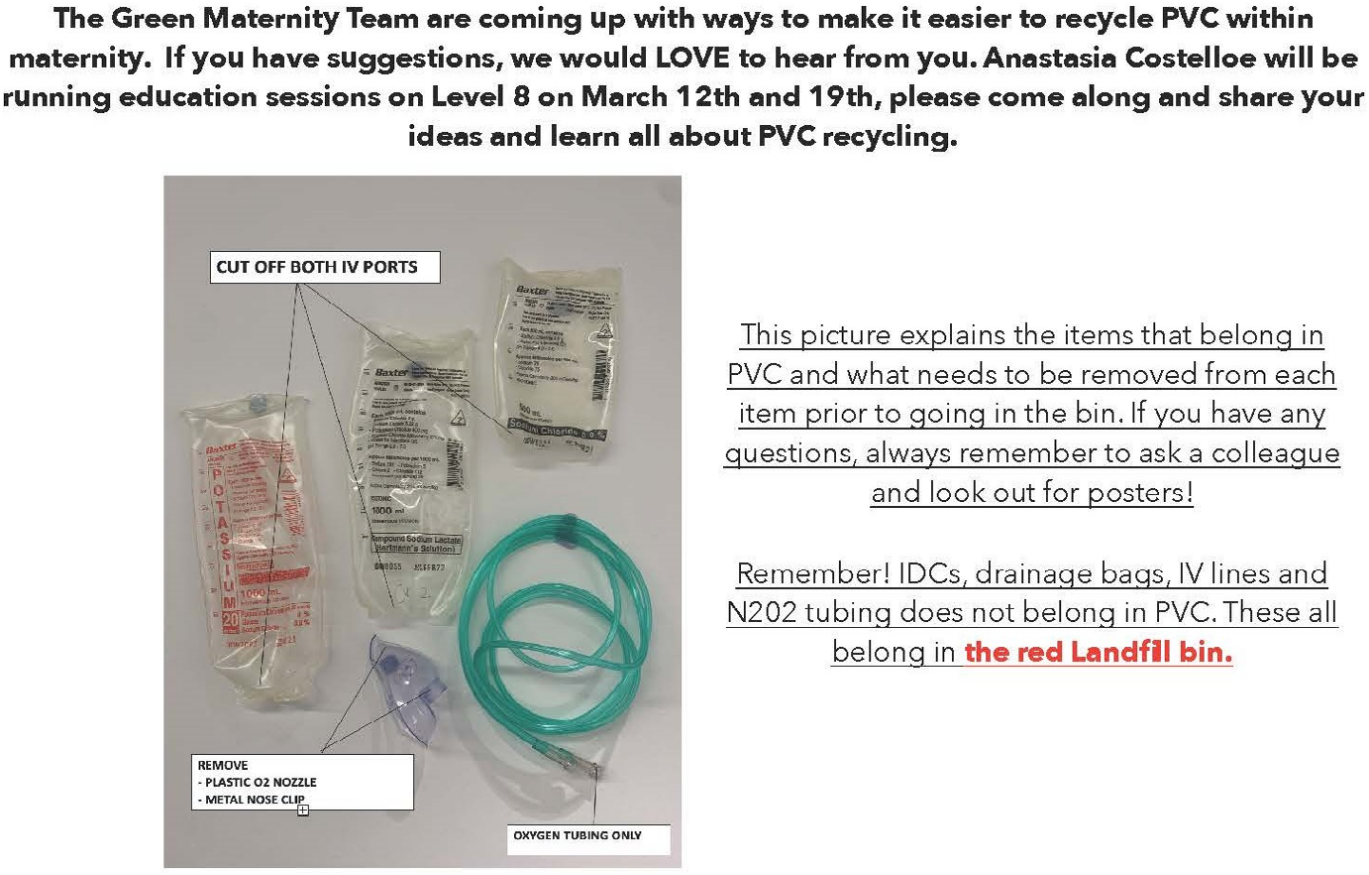
Image from monthly education newsletter.

The intervention commenced in January 2021 and was completed in December 2021. Instructional posters outlining adequate waste preparation for disposal (e.g. cutting of intravenous [IV] fluid bags) and appropriate waste segregation were placed near the bin locations (Figure 1). A monthly newsletter detailing key waste management strategies for the month and other waste/sustainability‐related information was distributed to staff using the maternity staff email list (Figure 2). Face‐to‐face and online education sessions about effective waste management in the postnatal ward were conducted in April, May, June and July 2021. Approximately 140 staff rotate through the postnatal ward at the study site. A total of 73 staff attended the available education sessions across the course of the intervention. Education sessions were also scheduled for August, September and October; however, they were not conducted due to COVID‐19‐related priorities at the hospital. Topics covered in the education sessions and the overall intervention are included in Table 1. Incentives to promote staff engagement with the initiative (such as prize draws) were also provided monthly. A total of 19 midwifery staff (that were not members of the research team) self‐nominated to be sustainability champions for the duration of the study and beyond. The sustainability champions were responsible for promoting best practices for waste management and segregation when working on the ward.

**TABLE 1:** Topics covered in the Green Maternity intervention.

PVC recycling
Clinical waste and sharps disposal
Co‐mingled recycling
Reducing waste in staff tea rooms
Waste segregation in administrative areas
Reducing baby care and feeding waste
Reducing waste in the medication room

### 2.2 Design

The study was an observational and cross‐sectional design. The study measures included pre‐ and post‐intervention surveys and observational waste collection audits.

#### 2.2.1 Sample and setting

The study setting was a postnatal ward of a large tertiary maternity service located in Melbourne, Australia. The maternity service manages over 6500 births per year. All clinical staff working in the postnatal ward of the service were invited to participate in the pre‐ and post‐intervention surveys during the data collection periods. This included midwives, nurses, managers, Registered Undergraduate Students of Midwifery (RUSOM) and lactation consultants. Approximately 140 clinical staff rotate through the postnatal ward; 59 completed the pre‐intervention survey and 65 completed the post‐intervention survey. We did not match pre‐ and post‐survey responses as staff rotate frequently across different maternity areas. The staff who were working on the postnatal wards at the time of the pre‐ and post‐surveys were likely to be different.

#### 2.2.2 Data collection

A descriptive staff survey was administered pre‐ and post‐intervention to measure knowledge and attitudes relating to waste management in the postnatal ward. A member of the research team piloted the survey with two midwifery collegues for comprehension and usability prior to distribution. The survey was administered using the online system REDCap and had 28 items, including:

- basic demographic questions;
- single‐ and multi‐response questions to determine waste management/segregation knowledge and attitudes (e.g. the definition of waste management, identifying what specific items go in what bin, perspectives regarding waste segregation and who should be responsible for doing it);
- four‐point Likert scale response questions with ratings from 1–4 strongly agree  to strongly disagree regarding personal waste management behaviour and opinions of the organization's approach to waste management;
- a single‐response question asking staff to rate the waste management and minimization performance (poor, good, excellent or I do not know) of the organization; and
- two final open‐text response questions to obtain suggestions for improving waste minimization at the organization and any further comments regarding waste management.

Staff were sent the pre‐intervention survey between February and March 2021 and the post‐intervention surveys between February and May 2022. The survey was distributed via the hospital staff email list for clinical maternity staff working in the postnatal ward. Consent was implied upon submission of the survey. Staff who completed the survey could opt to include an email address to enter a draw to win a prize worth $50AUD. The submitted emails were removed from the data to ensure the anonymity of responses.

Waste audit data were collected to measure the volume of waste within a 24‐h period at three time points in 2021, January, July and December. Waste volume in kilograms (kg) was recorded using a paper‐based waste audit record tool. Volumes of waste in each stream, including clinical, general and recyclable, were recorded. Comments related to specific observations (e.g. the presence of intravenous bags in clinical waste that could have been cut and disposed of in general waste) in each waste stream were also documented. Photographs of waste segregation were taken to document waste types in each stream. An occupational health and safety expert oversaw the waste audits to ensure the safety of the midwife researchers. Costs for managing each waste stream and total waste volume were calculated for each waste audit.

##### 2.2.2.1 Ethical considerations

Ethics approval was obtained from the hospital ethics committee (QA2020.114.72110). The participant information sheet was distributed with the survey link via staff email. As outlined in the participant information, consent was implied upon submission of the survey.

###### 2.2.2.1.1 Data analysis

The quantitative survey and waste audit data were analysed using SPSS software. Data were summarized as descriptive statistics (frequency and percentages). Pre‐ and post‐intervention survey data were analysed using chi‐square. Likert scale responses were dichotomized (agree vs. disagree) for analysis. Estimated cost savings post‐intervention were calculated to demonstrate the financial impact of the intervention. The actual cost for managing waste could not be shared publicly due to confidentiality agreements between the organization and their chosen waste management service. Qualitative survey data were analysed using content analysis (Bengtsson, 2016) in the software program NVivo.

###### 2.2.2.1.2 Validity, reliability and rigour

This study was guided by the Strengthening the Reporting of Observational Studies in Epidemiology guidelines. To our knowledge, no validated tools existed to measure knowledge and attitude of healthcare providers in relation to waste management and segregation. Our survey was developed based on review of similar research (Elsayed & Gab‐Allah, 2019; Runcie, 2018; Ryan et al., 2020; Sapkota et al., 2014; Slutzman et al., 2022; West et al., 2020; Wyssusek et al., 2016, 2019) and tools in the literature (Hames, 2013). We also had the support of sustainability experts to inform development of the survey, intervention, and data collection processes on the research team. The research team had experts in quantitative and qualitative analysis to support data analysis.

## 3 RESULTS

### 3.1 Pre‐ and post‐intervention staff survey quantitative data

#### 3.1.1 Demographic and employment characteristics

There were no significant differences between the pre (*n* = 59) and post (*n* = 65) intervention samples based on demographic/employment‐related characteristics. Most staff who participated in the surveys were aged between 26 and 35 years. All surveys were completed by midwives or nurses in the pre‐intervention phase and in the post‐intervention phase a doctor, a RUSOM and three management staff were included in the sample. Most staff (>85%) were employed at the hospital for 10 years or less. Only a small proportion of participants (*n* = 7) had received waste management training at their employment induction at the hospital.

#### 3.1.2 Definition of waste management

Almost all of the staff in the pre‐intervention survey (*n* = 56, 94.9%) and the post‐intervention survey (*n* = 63, 96.9%) correctly identified the definition of waste management, which was: ‘Before disposing of waste items, the staff member separates the items and disposes of them in the appropriate bin’. Two participants (3.4%) in the pre‐intervention survey incorrectly responded that waste management is defined as: ‘All waste transported out of the hospital is sorted and separated at an external location and disposed of appropriately’. A small proportion of participants indicated that none of the available options accurately defined waste management in the pre‐intervention (*n* = 1, 1.7%) and post‐intervention (*n* = 2, 3.1%) surveys.

#### 3.1.3 Knowledge of waste segregation

Participants were asked to identify the type of waste that went into the specific bin type. Most participants (over 85%) accurately identified the kind of waste that goes into each bin type in both pre‐ and post‐intervention phases (Table 2). They were also asked to select the correct waste stream for a particular item commonly found in the postnatal ward. Knowledge of accurate allocation of specific items to waste streams was variable amongst the group (Tables 2 and 3). The largest number of participants in pre‐ and post‐intervention periods correctly identified that a syringe used for IV antibiotics goes in a sharps container. However, a considerable proportion incorrectly determined that the syringe should go into general waste. Over 65% of participants in both periods correctly identified that a coffee cup should go into general waste. This was followed by approximately 30% of participants who incorrectly selected that it should go into recycling. The selection for the IV line showed the most variability, with around 30% of participants selecting it should go into general waste (correct), clinical waste and recycling, respectively. Most staff chose that the indwelling catheter should go into clinical waste (over 70%) when it should go into general waste, identified correctly by approximately 17% pre‐intervention and 23% post‐intervention. Almost all participants correctly determined that paper towel goes into general waste. Most correctly identified that a baby bottle should go into recycling, though a considerable proportion (over 20%) suggested it go into general waste. Statistical analysis showed no significant differences in knowledge of bin types and allocation of specific items to waste streams pre‐ or post‐intervention.

**TABLE 2:** Knowledge of waste types for bins available in the postnatal ward.

	Pre‐intervention *n* (%)	Post‐intervention *n* (%)
Red bin		
General waste^a^	52 (89.7)	59 (90.8)
Clinical waste	3 (5.2)	3 (4.6)
Sharps	1 (1.7)	0 (0.0)
Unsure	2 (3.4)	3 (4.6)
Blue bin		
Recycling^a^	53 (91.4)	57 (87.7)
General waste	3 (5.2)	0 (0.0)
Confidential waste	1 (1.7)	4 (6.2)
Unsure	1 (1.7)	4 (6.2)
Yellow bin		
Clinical waste^a^	52 (89.7)	56 (86.2)
Sharps	5 (8.6)	8 (12.3)
Recycling	1 (1.7)	1 (1.5)

^a^ Identifies correct response option.

**TABLE 3:** Knowledge of specific waste type disposal in appropriate bin.

	Pre‐intervention *n* (%)	Post‐intervention *n* (%)
Syringe used for IV antibiotics		
Sharps^a^	28 (47.5)	28 (43.1)
General waste	14 (23.7)	21 (32.3)
Clinical waste	8 (13.6)	10 (15.4)
Recycling	9 (15.3)	6 (9.2)
Coffee cup		
General waste^a^	39 (66.1)	46 (70.8)
Recycling	18 (30.5)	19 (29.2)
Unsure	2 (3.4)	0 (0.0)
IV Line		
General waste^a^	18 (30.5)	22 (33.8)
Clinical waste	17 (28.8)	19 (29.2)
Recycling	18 (30.5)	17 (26.2)
Anatomical	3 (5.1)	0 (0.0)
Sharps	1 (1.7)	1 (1.5)
Unsure	2 (3.4)	6 (9.2)
Indwelling catheter		
General waste^a^	10 (16.9)	15 (23.1)
Clinical waste	42 (71.2)	48 (73.8)
Recycling	1 (1.7)	0 (0.0)
Anatomical	4 (6.8)	2 (3.1)
Unsure	2 (3.4)	0 (0.0)
Paper towel		
General waste^a^	49 (83.1)	57 (87.7)
Recycling	10 (16.9)	6 (9.2)
Confidential waste	0 (0.0)	1 (1.5)
Unsure	0 (0.0)	1 (1.5)
Baby bottle		
Recycling^a^	42 (71.2)	44 (67.7)
General waste	13 (22.0)	19 (29.2)
Clinical waste	1 (1.7)	0 (0.0)
Unsure	3 (5.1)	2 (3.1)

^a^ Identifies correct response option.

#### 3.1.4 Opinions about staff performing waste segregation

Nearly all the staff in the pre (*n* = 58, 98.3%) and the post (*n* = 61, 93.8%) surveys reported that they believed it was the responsibility of staff to segregate waste during their employment at the hospital. One respondent in the post‐intervention survey identified that all hospital waste is clinical waste and should go in one bin, not requiring segregation. Two participants (3.1%) in the post‐intervention survey indicated that it is a waste of resources and time for staff to segregate waste into the required bins. Two participants, one in the pre‐intervention and one in the post‐intervention survey, did not identify with any of the opinion statements, selecting the ‘none of the above’ option.

#### 3.1.5 Reasons why staff do not segregate waste appropriately

Perceptions about inappropriate waste segregation were mostly consistent amongst pre‐ and post‐intervention surveys (Table 4). The most cited reason for staff not segregating waste appropriately was a lack of knowledge about waste types that go into each bin (over 75%). This was followed by time constraints (over 76%), bin location (over 49%) and lack of interest in waste disposal (over 39%). Only three staff reported that all staff segregate waste appropriately during their shifts on the postnatal ward.

**TABLE 4:** Perceived reasons for why staff do not segregate waste appropriately during their shifts.

	Pre‐intervention *n* (%)	Post‐intervention *n* (%)
Not knowing what goes into what bin	52 (88.1)	49 (75.4)
Time constraints	45 (76.3)	50 (80.0)
Bin location	32 (54.2)	32 (49.2)
Lack of interest in the effective waste disposal	23 (39.0)	33 (50.8)
All staff segregate waste appropriately	2 (3.4)	1 (1.5)

#### 3.1.6 Staff practices, attitudes and opinions of waste management at the organization

Staff rated their level of agreement on statements regarding waste management practices and their attitudes or opinions related to waste management and sustainability (Table 5). Overall, there were minimal differences between practices and attitudes to waste management in the pre‐ and post‐intervention surveys. Most participants agreed that they tried to reduce waste regularly and were concerned about the impact of the organisation's practices on the environment. A considerable proportion of staff in the pre (42.1%) and the post (58.5%) intervention groups disagreed that segregating waste in the correct bins was an easy task and did not impact their time management while working. Many participants in both groups disagreed that the organisation took waste minimisation seriously; however, there was a significant increase in in the proportion of participants who agreed that enough was being done in maternity services to minimise waste post‐intervention.

**TABLE 5:** Staff practices, attitudes and opinions of waste management at the organization.

Survey statement	Percentage agree	
	Pre‐intervention (*n* = 59)	Post‐intervention (*n* = 65)
	*n* (%)	*n* (%)
I regularly try to reduce waste and practice recycling	53 (96.4)	64 (98.5)
I find separating the waste into the correct bins is an easy task that does not impact my time management during work	33 (57.9)	27 (41.5)
I am concerned about the impact our organization has on landfill waste and the carbon footprint	54 (100)	61 (93.8)
I would attend a training session to increase my knowledge of waste segregation	51 (94.4)	60 (93.8)
We are doing enough in maternity services to minimize waste	5 (8.5)	15 (23.1)*
Our organization takes waste minimization and environmental sustainability very seriously	29 (49.2)	34 (52.3)
Our organization is proactive in addressing its waste management issues	29 (49.2)	37 (56.9)
The executives and management staff actively	24 (40.7)	23 (35.4)
Encourage environmental sustainability at our organization		

* Significant at *p* < .05.

#### 3.1.7 Perception of overall organisation waste minimization and management performance

Participants were asked to rate the organisation's performance on waste minimization and management practices. The majority of participants in the pre‐intervention period rated performance as poor (*n* = 28, 47.5%), followed by good (*n* = 18, 30.5%). The highest proportion of participants in the post‐intervention period rated performance as good (*n* = 35, 53.8%), followed by poor (*n* = 23, 35.4%). Only one participant in the pre‐ and post‐intervention periods rated performance as excellent. The remaining participants in the pre‐intervention period (*n* = 12, 20.3%) and the post‐intervention period (*n* = 6, 9.2%) indicated they were unsure about the organization's waste management performance.

### 3.2 Pre‐ and post‐intervention staff survey qualitative data

Qualitative responses to the survey asked participants to describe ways to improve waste minimization at the service and additional comments were analysed. The content analysis revealed the most common strategies for promoting effective waste management and segregation (Table 6). The most identified strategy was providing ongoing education about appropriate waste management and segregation using face‐to‐face and online delivery. This was followed by increasing the number and variety of bins available and improving bin locations for easy access. Clear signage identifying what items can go in what bin was frequently reported. Changing to sustainable/reusable materials for common items used on the wards and employing sustainable suppliers were mentioned as strategies to reduce waste and minimize the environmental impact. Increasing awareness about effective waste management by engaging staff to demonstrate and communicate best practices during their shifts was seen as a helpful strategy to improve waste minimization. Increasing recycling options so that other commonly used materials such as soft plastics and medication foil blister packs could be repurposed was also mentioned. Having tools available (particularly scissors) near clinical bin locations so that items could be effectively prepared for disposal (e.g. cutting IV bags) was suggested by several participants. Incorporating incentives to encourage effective waste management was identified as a strategy to increase appropriate practice. A few staff mentioned that increased workload impeded their ability to dispose of waste while they worked effectively.

**TABLE 6:** Content analysis of open qualitative survey responses.

Category name and number of comments	Illustrative texts
Ongoing waste management education (*n* = 52)	In service to remind staff of segregation of waste and what needs to be clinical waste vs general and recycling. Or online WeLearn package yearly with updates and refreshes also including the impact hospitals can have on pollution and negative impact the environment so staff are aware of how important this issue is and that we all have a role to play for our earth! (P50)
More bins and improving bin location (*n* = 25)	My biggest issue with correct waste management is the layout of the ward itself – because it is so long end to end, some days I am running back and forth and if the correct bin is not available, I cannot afford the time or energy it takes to locate the correct bin. I know it sounds lazy but the extra steps add up. I would truly recycle more if I did not have to go looking for the correct bins/locate scissors before cutting my iv bag. (P35)
Signage of what goes into what bin (*n* = 22)	Pictures/lists above bins of what can go in them. (P125)
Changing to reusable and sustainable products (*n* = 17)	People need to stop being so lazy and think that their little contribution makes no difference in the grand scheme… is does. Encourage manufacturers of medical products to use minimal packaging. Buy from suppliers who are like minded about sustainability… not just those with the best price. (P7)
	More education should be given to women about placing nappies in the appropriate waste bin. Engage with local artists and have them repurpose some of our appropriate recyclable items. Provide larger containers for collecting caps off vials. Provide separate containers for the baby bottles, lids, teats. I think the Green Team are doing a great job. (P118)
Increase awareness and demonstrate best practice (*n* = 14)	Create a culture of environmental friendly midwives. (P101)
	Staff being proactive and encouraging each other to ‘do the right thing’. (P7)
Provide easy access to tools required to complete effective waste management (*n* = 5)	Another problem is that the women only have general rubbish bins in there room it would be better to have another small recycling bin. Or in the milk room have a specific bottle recycling bin like at other hospitals. Also having scissors located in the pan rooms as well as the medication room would make it easier to cut iv lines and syringes and dispose correctly. (P42)
Improve recycling options (*n* = 5)	Increase amount of recycling options. Recycle blister packs, soft plastics, glass vials etc. (P48)
Provide incentives for effective waste management (*n* = 3)	I think the hsopital tries by providing the bins in all the right places but every bin I see has incorrect items in them. I think some staff dont care and others just dont know which bins. I think more education is needed. Maybe even some incentives such as financial benefit to wards that recycle well. Such as increased equipment. (P33)
Workload impedes effective waste management (*n* = 3)	As a staff member I find myself so conscious at home to reduce waste, but personally I struggle at work due to the huge work load and lack of desire from other staff members. We're so overworked waste reduction is one of the last things on my mind. (P100)

### 3.3 Waste audit data

#### 3.3.1 Waste volume by type

**FIGURE 3:**
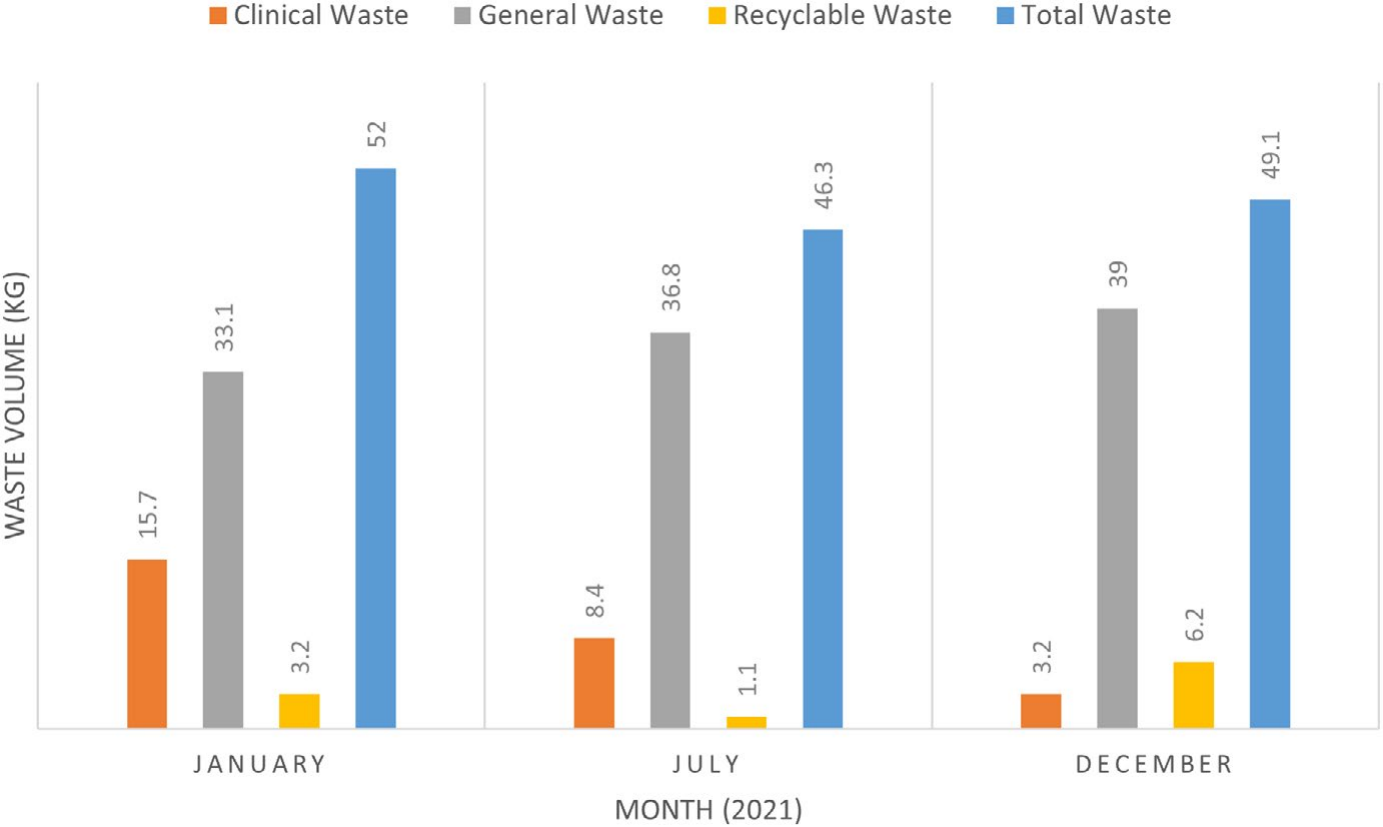
Audit of waste volume from commencement to the end of the intervention.

The total volume of waste (kg) changes in each waste stream and total waste from the commencement of the intervention (January) to the end (December) is presented in Figure 3. The waste audit data showed that reductions in clinical waste occurred at each time point post‐intervention. Table 7 shows a 71.2% decrease in clinical waste from baseline to the final post‐intervention audit in December. General waste increased at each time point, increasing by 17.8% at the final audit. Recycling volume dropped from baseline to the mid‐intervention audit in July. However, it increased by 93.8% when comparing the baseline to the final audit in December. Each waste stream's proportion of different waste types was also measured. The percentage of clinical waste observed in the general waste stream increased from baseline (6.6%) to mid‐intervention audit in July (13.8%) and then dropped back to slightly below baseline level at the final audit (6.2%) in December. The proportion of general waste in the recyclable waste stream increased at each audit point in July (27.3%) and December (48.4%) compared to the baseline (9.4%). In comparison to the initial audit of the proportion of recyclables in the general waste stream (9.1%), slight increases were seen in July (10.3%) and December (12.8%) post‐intervention.

**TABLE 7:** Audit of waste volume and proportion of different waste types in each waste stream.

		Proportion of waste type in each waste stream				% Change of total waste volume from baseline
Month	Waste stream	Clinical kg (%)	General kg (%)	Recyclable kg (%)	Total kg (%)	
January (baseline)	Clinical^a^	15.7	‐	‐	15.7 (30.2)	
	General	2.2 (6.6)	27.9 (84.3)	3.0 (9.1)	33.1 (63.6)	
	Recycling	0.0 (0.0)	0.3 (9.4)	2.9 (90.6)	3.2 (6.2)	
	Total				52.0 (100.0)	
July	Clinical^a^	8.4	‐	‐	8.4 (18.1)	−46.5%
	General	5.1 (13.8)	27.9 (75.8)	3.8 (10.3)	36.8 (79.5)	+11.2%
	Recycling	0.0 (0.0)	0.3 (27.3)	0.8 (72.7)	1.1 (2.4)	−61.3%
	Total				46.3(100.0)	−11.0%
December	Clinical^a^	3.9	‐	‐	3.9 (8.0)	−71.2%
	General	2.4 (6.2)	31.6 (81.0)	5.0 (12.8)	39.0 (79.4)	+17.8%
	Recycling	0.1 (1.6)	3.0 (48.4)	3.1 (50.0)	6.2 (12.6)	+93.8%
	Total				49.1 (100.0)	−5.8%

^a^ Composition could not be observed due to health and safety requirements.

#### 3.3.2 Visual assessment of waste segregation

During each audit, items incorrectly placed in bins were identified. At baseline, there were many indwelling catheters and whole IV lines and bags incorrectly placed in clinical waste, confidential information and linen in general waste, and styrofoam cups in recyclable waste. In the July audit, there was a similar pattern with indwelling catheters and whole IV lines and bags in the clinical waste, confidential information, IV bags, lancets, and linen in general waste, and styrofoam cups and food in the recyclable waste. During the final audit, there remained some indwelling catheters and whole IV lines and bags incorrectly placed in clinical waste although this had significantly reduced. There remained confidential information, face masks, and lancets in general waste (but no linen), and styrofoam cups and food in recyclable waste. Amounts of food in the recycling bins, however, decreased substantially over time.

#### 3.3.3 The cost impact of the intervention

The cost of managing the waste was reduced at each audit post‐baseline. From January to July, the cost decreased by 30.4%. When comparing the January audit to the final audit in December, the cost was further reduced by 48%. The major cost savings were in the reduction of clinical waste through correct segregation.

## 4 DISCUSSION

The overall aim of the study was to determine the impact of a midwife‐led intervention to promote effective waste management and segregation in the postnatal ward in an Australian maternity hospital. Effects of the intervention were measured by changes in staff knowledge and attitudes regarding waste management and waste collection audits conducted at baseline, 6 and 12 months post‐implementation. The findings showed no significant differences in knowledge and minimal changes in attitudes post‐intervention; however, it is likely that the respondents were different and therefore true comparison of pre‐ and post‐intervention outcomes was not possible. Nearly all staff could correctly identify the different bin types available on the ward; however, there were more errors in understanding how to dispose of specific items. For example, many staff incorrectly nominated the indwelling catheter to be disposed of as clinical waste when it could be safely disposed of in the general waste. This highlights the need to continually educate staff about items placed in clinical waste bins incorrectly. Instructional signage near bins to support appropriate segregation was valued by participants in the study and has been identified as a critical factor for supporting behaviour change (Hames, 2013). An analysis of maternal/child health nurses showed significant increases in waste management knowledge following targeted education (Elsayed & Gab‐Allah, 2019) as was done in the present study.

Most participants agreed they would be willing to attend waste management education both pre‐ and post‐intervention. The number of participants who attended education available during the intervention was considerable; however, changes in practice due to COVID‐19 meant that many sessions could not go as planned or staff could not participate due to increased work demand, limiting the reach of the education. This may have resulted in the minimal change in pre‐ and post‐knowledge scores. Despite no changes in knowledge, the observational audits showed that there was correction of segregation practices over time. One explanation is that staff who had greater knowledge and were segregating effectively may not have completed the survey. Alternatively, behaviour change may have been influenced by other factors irrespective of knowledge. Social research suggests that social modelling (Perry et al., 2021) (e.g. the midwife champions) and cues to action (e.g. instructions and waste preparation tools near bins) have much stronger influences on pro‐environmental behaviour change than knowledge or education (Bergquist et al., 2019; Linder et al., 2021, 2022). Many survey respondents did however indicate that implementing education sessions to serve as reminders for accurate waste segregation would be beneficial. They also highlighted bin location as a key barrier to effective waste segregation. Research has shown that education has limited influence on behaviour (McKenzie‐Mohr & Schultz, 2014); however, combining this with cues to action and improving bin location is likey to result in improved waste segregation practices (Linder et al., 2021).

More participants agreed post‐intervention that the hospital was being proactive in reducing waste, which may be due to the visibility of the Green Maternity intervention on the ward. Continued action to ensure initiatives are visible to staff can promote ongoing efforts to minimize waste (Wyssusek et al., 2019).

Consistent with other healthcare waste management interventions (Ashtari et al., 2020), the results of the post‐intervention waste audit demonstrated the greatest change, with considerable clinical waste reduction and increased recyclable waste. Costs of managing waste were almost halved when comparing pre‐ and post‐intervention audits. This demonstrates that despite the minimal impact on knowledge and attitude, the various initiatives to promote better waste management did encourage behaviour change and resulted in better waste management practices. Given that clinical waste is approximately five times the cost of general waste, this audit shows a significant cost saving to the organization. The hospital is predicted to produce approximately 56 tonnes of clinical waste annually (Western Health, 2022), incorporating interventions such as this across the organization are likely to be associated with substantial economic and environmental benefits.

Reports suggest that without effective leadership, waste management behaviour change is difficult to achieve (Wyssusek et al., 2019). The leadership of the Green Maternity midwives, alongside the initiatives throughout the ward to encourage compliance with effective waste segregation, are likely to have resulted in in improved waste segregation over time. Comments on the surveys were highly supportive of the Green Maternity team, suggesting that staff value waste management ‘champions’ who demonstrate best practices and encourage compliance. Rolling out similar initiatives across hospital wards have the potential to significantly reduce incorrect waste management practices that produce scope 3 carbon emissions. This is likely to be supportive of efforts to meet Australian carbon emission targets.

### 4.1 Limitations

The study is strengthened by a good response rate (43%) from staff to the surveys, despite the pressures of the pandemic and the increasing birth rate at the hospital. As the survey was voluntary, more participants interested in waste management may have completed it, increasing the risk of bias. Having the same participants complete the survey pre‐ and post‐intervention would have enhanced the results; however, due to staff rotations across the maternity wards, this was not possible. The waste audits were conducted using one‐off 24‐h collections at each time point. To avoid influencing staff behaviour, the dates of these audits were not known by the ward staff. A greater number of measurements to ensure that other factors did not contribute to waste disposal could improve the reliability of the results.

## 5 CONCLUSION

The midwife‐led Green Maternity initiative improved waste segregation and associated waste management cost reduction. Inadequate waste minimization and management by healthcare services are likely to lead to significant environmental impact. With appropriate support, midwives can drive behavioural changes that can reduce the carbon footprint of maternity wards.

## FUNDING INFORMATION

The study was supported by funds provided by a Western Health Foundation staff research grant.

## CONFLICT OF INTEREST STATEMENT

The authors declare no conflict of interest in relation to this study.

## STATISTICS

That authors affirm that the methods used in the data analyses are suitably applied to their data within their study design and context, and the statistical findings have been implemented and interpreted correctly.

## Data Availability

The data that support the findings of this study are available on request from the corresponding author. The data are not publicly available due to privacy or ethical restrictions.
